# Incorporating the Psychological Perspective into Traditional Prevention in Pediatric Dentistry with the PaFein+ Project: Trained Parents Can Help

**DOI:** 10.3390/bs16040620

**Published:** 2026-04-21

**Authors:** Aneta Munteanu, Arina Vinereanu, Ruxandra Sfeatcu, Mihaela Tănase, Ilie-Andrei Condurache, Annelyse Garret-Bernardin, Alessandra Putrino, Özgür Önder Kușçu, Sertac Peker, Betul Kargul, Angela Galeotti

**Affiliations:** 1 Pedodontics Department, Faculty of Dentistry, Carol Davila University of Medicine and Pharmacy, 041292 Bucharest, Romania; aneta.munteanu@umfcd.ro (A.M.); mihaela.tanase@umfcd.ro (M.T.); 2 Oral Health and Community Dentistry Department, Faculty of Dentistry, Carol Davila University of Medicine and Pharmacy, 041292 Bucharest, Romania; ruxandra.sfeatcu@umfcd.ro; 3 Dr Victor Babeș Clinical Hospital of Infectious and Tropical Diseases, 030303 Bucharest, Romania; ilie.condurache@spitalulbabes.ro; 4 Dentistry Unit, Management Innovations, Diagnostics and Clinical Pathways, Bambino Gesù Children’s Hospital, IRCCS, 00165 Rome, Italy; annelyse.garret@opbg.net (A.G.-B.); alessandra.putrino@opbg.net (A.P.); angela.galeotti@opbg.net (A.G.); 5 Pedodontics Department, Istanbul Kent University, 34433 Istanbul, Türkiye; ozguronder.kuscu@kent.edu.tr; 6 Pedodontics Department, Marmara University, 34854 Istanbul, Türkiye; sertacpeker@marmara.edu.tr (S.P.); bkargul@marmara.edu.tr (B.K.)

**Keywords:** dental anxiety, pediatric dentistry, parent education, health behavior

## Abstract

Background: Emotional aspects of early dental experiences have long-lasting effects. This study aimed to assess parents’ childhood dental experiences and their impact on current attitudes toward dental treatment and to evaluate the subjectively perceived usefulness of an educational material focused on psychological management of children’s dental visits. Methods: A cross-sectional descriptive pilot study was conducted using an educational booklet developed and distributed to parents, who read it and completed a short questionnaire. Responses received between 27 February–31 March 2025 were analyzed using IBM SPSS Statistics 25. Results: A total of 142 parents (88% mothers) participated. Most participants (83.1%) had a university degree. Negative childhood dental experiences were reported by 44.4% (more frequent among mothers, *p* < 0.001), and 18.3% had shared these experiences with their children. Emotional discomfort during dental visits was reported by 61.3% of respondents. Dental anxiety was significantly associated with negative childhood experiences (*p* < 0.001). Parents with higher education were more likely to choose a certified paedodontist for their child than a nearby general dentist (*p* = 0.002). Most parents (97.9%) found the material provided helpful for managing future dental visits, and 91.6% were willing to share it with others. Conclusions: Childhood dental fear and anxiety may persist into adulthood. Despite the limited generalizability of our results, parents appreciated targeted resources which may support them in promoting positive dental experiences for their children.

## 1. Introduction

### 1.1. Background

Dental fear and anxiety (DFA) are common conditions among children and adolescents worldwide, with the highest prevalence observed in preschoolers and school-aged children (Grisolia et al., 2021). However, DFA is often underestimated in terms of its occurrence, underlying causes, and—most importantly—its long-term consequences. When it develops early in life, DFA does not necessarily diminish with age. Negative perceptions of dental care may persist and significantly affect oral health throughout life, as individuals with DFA tend to postpone or even avoid dental visits altogether (Oliveira et al., 2017; Dahlander et al., 2019). Consequently, children experiencing DFA often present with higher caries experience, a greater number of untreated carious lesions, and poorer oral health-related quality of life compared to their peers without dental anxiety (Raadal et al., 2002; Porritt et al., 2016; Marshman et al., 2023).

Fear does not always develop as one’s conditioned response to direct exposure to a traumatic event. Indirect mechanisms, requiring no personal experience of the aversive event, may account for fear occurrence and key psychological frameworks for indirect fear transmission are described in the literature. Social learning and observational learning theories (Olsson et al., 2007; Emerson et al., 2019) suggest that fear can be learnt by observing the emotional reactions of others, being highly relevant in parent-to-child transmission, where children quickly adopt parental fears. Rachman (Rachman, 1977) identified and described three main pathways for fear acquisition: direct conditioning, vicarious acquisition, and transmission of information, with the latter two being indirect forms of transmission. Ollendick and King (1991) found that the majority of the studied children attributed the onset of their fears to vicarious and instructional factors rather than to direct conditioning.

Cognitive-behavioral models of childhood anxiety further highlight the role of parental cognitions and behaviors, suggesting that verbalizing potential threats, expressing anxiety, and engaging in overprotective or controlling parenting may contribute to the development of anxiety in children (Emerson et al., 2019). These indirect mechanisms help explain the intergenerational transmission of various forms of fear and anxiety, and dental fear is no exception.

Since parents serve as their children’s first and most influential role models from an early age, their approaches and attitudes toward health-related behaviors inevitably shape their children’s perceptions and habits, including those related to oral health. Studies highlight the need to increase parents’ awareness about preventing and promptly treating oral diseases in children (Kaushik & Sood, 2023; Doğan et al., 2025/2026; Erdede et al., 2022) while behavioral guidance (BG) receives less attention in both research and educational initiatives. Parenting has evolved considerably over time, continually adapting to societal changes. Public access to information—whether evidence-based or not—has expanded immensely. Dental professionals are responsible for guiding families in identifying and properly applying accurate information to ensure the greatest long-term benefit for their children’s health. This responsibility has traditionally been associated with the prevention of oral diseases; however, given the reasons previously discussed, dental fear and anxiety (DFA) should also be regarded as an essential component of preventive dentistry.

Parental engagement in establishing a positive, stress-free relationship between the child and the dental team plays a crucial role in the long term. Nevertheless, for all the mentioned reasons, simply encouraging parental involvement may not be sufficient. Structuring and supporting parents’ participation by educating them on how to effectively prepare for and manage their children’s dental visits can be highly beneficial, and initiatives aimed at fostering such competence should be actively developed (Bagattoni et al., 2022).

Pre-appointment parental counseling has been demonstrated to be highly effective. When parents are adequately informed about the procedures their child will undergo and about appropriate communication strategies—such as using clear explanations, demonstration videos, or illustrative drawings—pediatric dental patients tend to exhibit lower levels of fear and anxiety. This results in greater cooperation during dental treatment (Bagattoni et al., 2022; Ramesh et al., 2024).

The *Erasmus+ PaFein+* project seeks to cultivate generations of children with healthy dentition and positive attitudes toward oral health by promoting positive dental experiences, preventing the early development of DFA, and reducing the need for sedation or general anesthesia (GA)—thereby naturally generating an additional positive environmental impact. In line with these objectives, providing parents and families with appropriate, evidence-based information to help them prepare for and manage their children’s dental visits in a psychologically supportive and stress-reducing manner was identified as a key strategy for enhancing long-term preventive dentistry.

To address this need, a dedicated Parents’ Section was developed within the *PaFein+* project framework, and the present study forms part of the research conducted under this section.

### 1.2. Objectives

The aim of the study was to evaluate parents’ own childhood dental experiences and their potential influence on current perceptions and attitudes toward dental treatment. Additionally, the study assessed parents’ subjective perceptions of the effectiveness of the educational materials developed within the project to support them throughout their children’s dental care journey. This pilot study was designed to determine the acceptability, feasibility, and preliminary outcomes of the educational initiative prior to its potential expansion on a larger scale. In line with the study objectives, we proposed the following hypotheses: 1. Adverse dental experiences in childhood are likely to influence attitudes toward dental treatment in adulthood. 2. Parents demonstrate a need for educational interventions designed to strengthen their ability to foster supportive and positive dental experiences for their children.

## 2. Materials and Methods

### 2.1. Study Design

**Figure 1:**
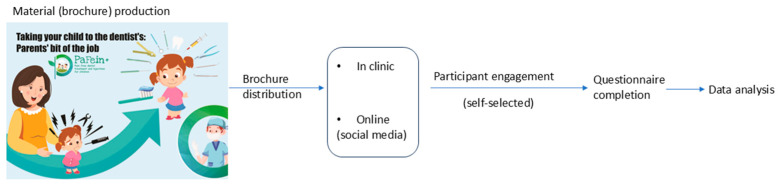
Project flowchart.

This was a cross-sectional, descriptive pilot study (Figure 1). In the first phase of the initiative, an informational booklet offering guidance on how to effectively prepare for and manage a child’s dental visit was developed by an interdisciplinary team of pediatric dentists and trained psychologists. The booklet provides practical advice for supporting the child before, during, and after the dental appointment, aiming to help families naturally integrate these experiences into everyday life and thereby minimize potential stress for all parties involved—the child, the family, and the dental team. To enhance understanding and applicability, the material was written in clear, accessible language and included examples of recommended and non-recommended wording to promote more effective communication.

### 2.2. Setting

**Figure 2:**
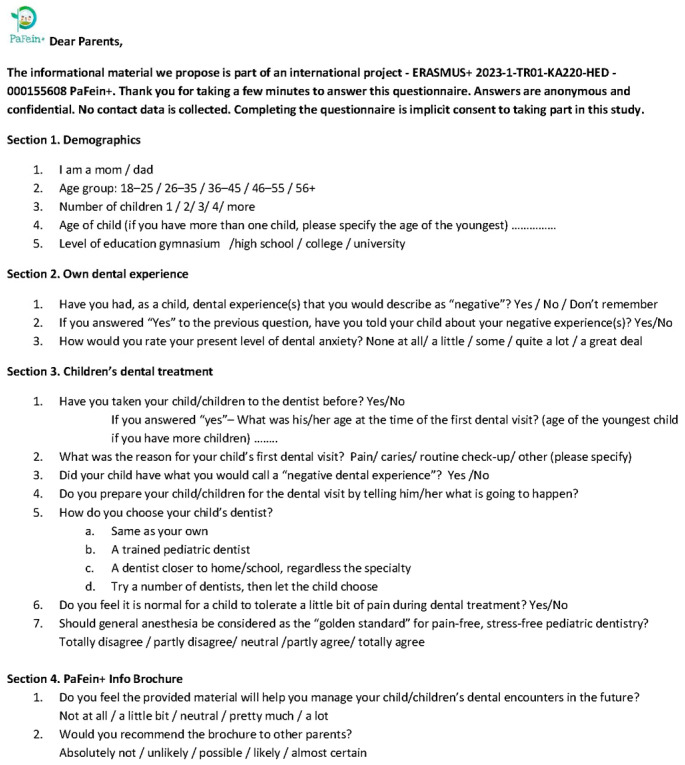
Questionnaire.

The educational material was distributed both digitally, via social media platforms, and in printed form (brochures) within the Pedodontics Clinic of the Carol Davila University of Medicine and Pharmacy in Bucharest, Romania. Parents who accessed the material were subsequently invited to complete a feedback questionnaire (Figure 2). The questionnaire was newly developed as a straightforward assessment tool and was validated only within the project team for use during this pilot phase. The average time for questionnaire completion was estimated to be 4–5 min. Parents’ answers were collected between 27 February–31 March 2025.

### 2.3. Participants

Eligibility criteria: Participation was voluntary and based on self-selection from parents who had accessed the contents of the informative material and wished to give their feedback. No additional inclusion criteria were applied; consequently, no responses were excluded.

Completion of the questionnaire specifically implied informed consent, and all responses were collected anonymously.

### 2.4. Bias

The subjective nature of answers, recall bias and potential social desirability were considered as potential sources of bias. While the first two factors cannot be counteracted, the anonymization of responses was expected to minimize the third.

### 2.5. Study Sampling and Sample Size

A minimal sample size was calculated using Gpower 3.1.9.7. Considering that most of the tested associations implied contingency tables between demographic parameters (such as gender, level of education (non-academic vs. academic)) or existence of possible childhood negative experience and other parameters analyzed in the study, based on an effect size of 0.3, an alpha probability of 0.05, minimal power of 0.8 and maximum degrees of freedom of 3, the calculated sample size was 122. The final sample size included in the study was 142, which would validate the initial study design.

### 2.6. Variables and Data Sources

The questionnaire was divided into 4 sections:

- Section 1 collected demographic information (parent gender, age group, number of children, age of children, level of education);
- Section 2 included three questions addressing the parents’ own childhood dental experiences and current attitudes toward dental care. Childhood dental experiences subjectively recalled as negative—defined as “dental experiences that you would describe as negative”—were recorded using a Yes/No/Don’t remember scale. The extent to which parents shared such negative experiences with their children was also assessed. To maintain simplicity in the questionnaire, participants’ current self-perceived level of dental anxiety was measured by a single-item self-report measure, using a five-point Likert scale (“none at all/a little/some/quite a lot/a great deal”);
- Section 3—explored parental attitudes toward their children’s dental treatment (previous dental visit with children, reason for dental visit, recalled children’s negative dental experience, preparation of children for dental visit, opinion about dental pain tolerance in children, opinion about general anesthesia as gold standard, preferred criteria for selecting a dentist for their children).
- Section 4 assessed the subjectively perceived usefulness of the educational material provided (opinion regarding helpfulness of PaFein+ brochure, level of recommendation to other parents).

Except children’s age (which is a quantitative variable), all other analyzed parameters were binomial/nominal qualitative variables.

### 2.7. Statistical Methods

Data was analyzed using IBM SPSS Statistics 25 and illustrated using Microsoft Office Excel/Word 2024. Children’s age (quantitative variable) was tested for normal distribution using the Shapiro–Wilk test and was written as averages with standard deviations or medians with interquartile ranges. Children’s age was tested between groups using the Mann–Whitney U Test/Kruskal–Wallis H Test. Qualitative variables were written as counts or percentages and were tested between groups using Fisher’s exact test. Z-tests with Bonferroni correction were used to further detail the results obtained in the contingency tables. Cramer’s V (ϕc) effect sizes were detailed for each contingency table comparison. The significance level threshold considered for all tests was α = 0.05.

## 3. Results

A number of 142 parents completed the feedback questionnaire. Table 1 presents the demographic characteristics of the participating parents (Section 1 of the questionnaire) together with their responses to Sections 2 and 3, which focused on childhood dental experiences and attitudes toward children’s dental care.

### 3.1. Demographic Data

Most participating parents (88%) were mothers; highly educated parents prevailed. The age of their children at the time of their first dental visit varied between 3 and 6 years.

### 3.2. Parents’ Own Childhood Dental Experiences and Current Level of Dental Anxiety

Negative childhood dental experiences were recalled and reported by 44.4% of parents. Z-tests with Bonferroni correction indicated that mothers were significantly more likely to recall negative experiences (*p* < 0.001, ϕc = 0.322), and 18.3% of participants reported having shared these experiences with their children.

**Figure 3:**
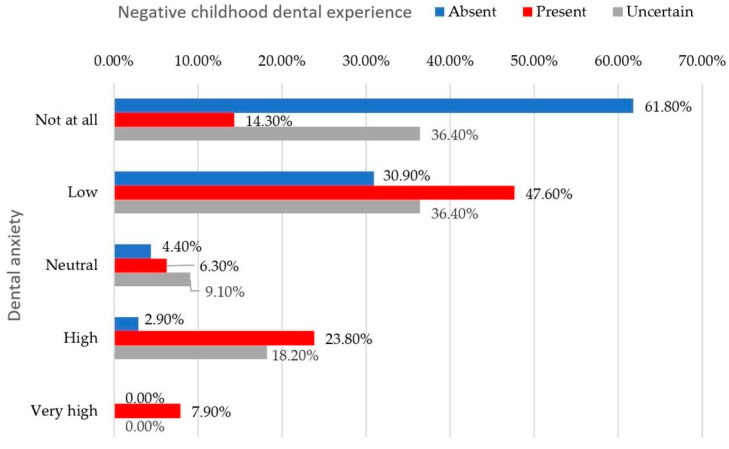
Distribution of participants based on the presence or absence of negative childhood dental experiences in relation to self-reported levels of dental anxiety (*p* < 0.001, Fisher’s exact test).

A total of 38.7% of respondents stated that they experienced no unease related to dental visits, whereas 61.3% acknowledged varying degrees of emotional discomfort The self-reported level of dental anxiety was significantly associated with negative childhood dental experiences (Fisher’s exact test, *p* < 0.001, ϕc = 0.371) (Figure 3).

### 3.3. Attitudes Toward Children’s Dental Treatment

The most frequently reported reason for visiting the dentist was a routine check-up (54.5%), followed by the presence of a noticed cavity (24.2%).

**Figure 4:**
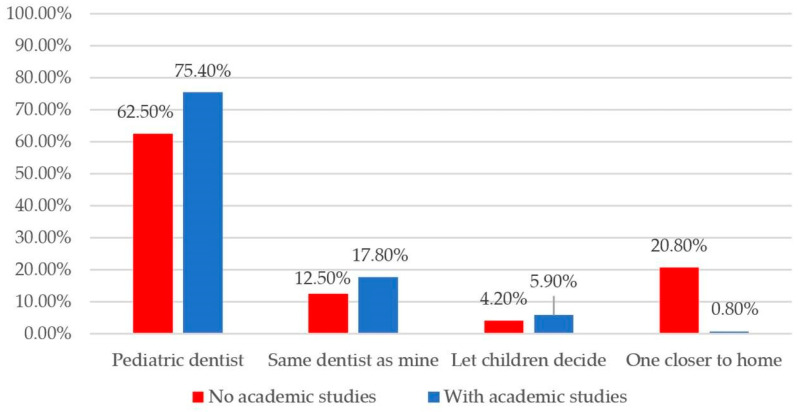
Distribution of participants according to their level of education and preferred criteria for choosing a dentist for their child.

Most parents (73.2%) preferred their children to be treated by a trained pediatric dentist. Differences in the selection criteria for a child’s dentist were significantly influenced by the parents’ level of education. Parents without academic qualifications were significantly more likely to base their choice on geographic proximity rather than professional specialization, preferring a dentist located closer to their home (20.8% vs. 0.8%; *p* = 0.002, ϕc = 0.373) (Figure 4).

Most responding parents (85.2%) reported that they prepared their children for dental visits in advance. Most parents (66.9%) considered it normal for a child to experience some level of pain during dental treatment, with fathers being significantly more likely to agree with this statement than mothers (*p* = 0.001, ϕc = 0.259). Additionally, 14.1% of parents believed that GA should represent the gold standard for ensuring stress- and pain-free pediatric dental care. However, this perception was not correlated with the parents’ self-reported level of dental anxiety (Table 2). Similarly, parents’ tendency to share stories about their own negative childhood dental experiences with their children was not significantly associated with their degree of dental anxiety (Table 2).

### 3.4. Perceived Usefulness of Provided Information

Most respondents (97.9%) felt that the provided educational material would help them, to varying degrees, better manage their children’s future dental visits (44.4% responded “a lot”; 28.2% responded “very much”). Additionally, 91.6% indicated that they would share the material with other parents or families (54.2% responded “very high level of agreement”; 37.3% responded “high level of agreement”) (Table 3).

## 4. Discussion

### 4.1. Key Results

Our study identified a significant correlation between adverse dental experiences during childhood and the respondents’ attitudes toward dental treatment in adulthood. Parents expressed a positive reception toward the educational intervention aimed at enhancing their capacity to promote supportive and positive dental experiences for their children. These results sustain the hypotheses initially proposed in the study.

The calculated mean age of the respondents’ children at the time of their first dental visit (4.45 ± 2.3 years) is rather high from a preventive perspective, aligning with previous reports (Besiroglu-Turgut et al., 2024). Similar ages at the first dental visit have also been documented (Padung et al., 2022), despite international professional organizations recommending that children have their first dental visit by 12 months of age to establish a “Dental Home” (DH) as early as possible (Nowak & Casamassimo, 2002). The advantages of the DH system are well recognized and have been proven over time, both for healthy children and for those with special healthcare needs (Nowak & Casamassimo, 2002). Among its most important benefits is the absence of dental fear, which is a key characteristic of this care model.

Two-thirds of the respondents (66.9%) showed a clear tendency to accept some degree of pain associated with their children’s dental treatment. This rather surprising perception may stem from cultural traditions that associate dental treatment with suffering and from parents’ own childhood experiences when access to advanced pain management was likely more limited. The observation that fathers were significantly more inclined than mothers to endorse this view may also reflect culturally rooted patterns, with fathers exhibiting a greater propensity toward stricter approaches to child discipline.

Contrary to our findings, a recent systematic review on parents’ perceptions of dental interventions for their children identified practitioners’ ability to manage pain and fear as a key factor in treatment acceptance (Dalsochio et al., 2025).

Previous studies have shown that children who are psychologically prepared for dental visits exhibit lower levels of anxiety (Bagattoni et al., 2022; Fox & Newton, 2006; Freeman, 2007). Such preparation may be directed at children themselves—in the waiting room (Fox & Newton, 2006; Freeman, 2007)—or indirectly, through parental guidance (Bagattoni et al., 2022; Porritt et al., 2013). A controlled trial by Fox and Newton (2006) demonstrated that exposing children to positive images of dentistry before their appointment fosters a more positive relationship with the dentist by temporarily reducing anticipatory anxiety. However, anticipatory anxiety showed no correlation with age or gender (Fox & Newton, 2006).

Another potential approach is to train parents—through booklets, videos, smartphone applications, and similar tools—on effective strategies for preparing their children for dental visits (Bagattoni et al., 2022; Porritt et al., 2013). Bagattoni et al. (2022) demonstrated the effectiveness of this kind of intervention; in their study no operative or invasive procedures were performed during the first dental encounter. The findings demonstrated that children whose parents implemented the booklet’s recommendations reported lower levels of pain and perceived the visit as more enjoyable compared to children whose parents had not received the material. It is essential that such psychological preparation takes place in a safe environment and is facilitated by an individual who is emotionally significant to the child (Bagattoni et al., 2022).

Mittal et al. (2025) conducted a randomized controlled trial to evaluate the impact of a specially developed dental modeling story on dental anxiety and behavior management among children aged 6–8 years. Its effects were compared with those of a traditional folk story. Both storytelling interventions resulted in significant reductions in anxiety, with no statistically significant differences between the two groups. Nevertheless, parents reported a preference for the dental modeling story (Mittal et al., 2025).

Interventions such as comic strips, booklets, and similar educational materials designed to inform children about dental treatment and visits are cost-effective, simple to administer, and can elicit positive behavioral outcomes (Bagattoni et al., 2022; Deshpande et al., 2002; Murshid, 2017; Meshki et al., 2018). For optimal effectiveness, parental guidance for preparing children for dental visits should primarily target parents of young children with no prior dental experience. Although taking their children to the dentist was not an inclusion criterion, all participants in our study reported previous dental encounters. Encouraging parents to access targeted resources prior to their children’s first dental visit would be recommended in order to increase the effectiveness of such educational initiatives, as children who experience negative emotions during their first dental visit are at increased risk of developing dental DFA (Bagattoni et al., 2022).

In the present study, most participating parents (over 85%) from the *PaFein+* project reported that they had prepared their children for dental appointments in advance. Nearly all respondents (97.9%) believed that the provided materials would enhance their ability to manage their children’s dental experiences in the future, and many (over 90%) indicated their willingness to share the information with peers. The project team considered these findings encouraging. Consequently, educational resources for families are being further diversified and refined in response to participant feedback. In line with prior research suggesting that parents perceive storytelling videos as relatable and reflective of authentic experiences (Dalsochio et al., 2025), an original educational video illustrating appropriate and inappropriate methods of supporting children before, during, and after dental visits was developed. The video depicts a real-life-inspired scenario of a five-year-old child’s first dental appointment, covering the sequence from entering the waiting room to the completion of treatment. It is organized into five distinct chapters, each highlighting specific “dos” and “don’ts.” The video is currently freely available in English and Romanian on YouTube (English version: https://www.youtube.com/watch?v=SBTjpmGo5k0; accessed on 13 January 2026; Romanian version: https://www.youtube.com/watch?v=QgCeQBw470M; accessed on 13 January 2026).

Further activities are planned, and the involvement of new potential partners—such as national pediatric dentistry societies, universities, parent groups and associations, and technology providers—is being sought and encouraged to support continued development of project content and to enhance its overall impact. Based on the results of this pilot study, the *PaFein+* educational initiative for parents will be further expanded in terms of both contents and geography, not only to the other countries participating in the project but also to any interested regions.

### 4.2. Limitations

This descriptive study presents certain limitations. Owing to its cross-sectional design, it cannot evaluate long-term effects on children’s behavior and cooperation in dental settings. Future longitudinal studies are required to assess the sustained efficacy of the educational materials provided to parents.

The participants in this study were self-selected, and most (88%) were mothers, while 83.1% were highly educated. Therefore, the findings should be generalized with caution. Potential recall bias, together with the inherently subjective interpretation of one’s childhood memories and potential social desirability bias, may constitute additional limitations to the study’s objectivity.

Feedback was obtained exclusively from parents, whereas the ultimate beneficiaries of these educational interventions are the children themselves. Objective measures are required in future research to evaluate the actual impact of parental educated involvement on children’s behavior. Moreover, the practical effectiveness of the materials provided could not be objectively verified, as the perceived usefulness of the booklet was based solely on parents’ subjective assessments.

Challenges were encountered in collecting feedback, particularly when parents accessed the materials through online or media channels. A larger number of respondents would allow for a more accurate and representative evaluation of the materials’ usefulness and perceived impact.

## 5. Conclusions

Childhood dental fear does not necessarily diminish with age. Parents value initiatives that may empower them to guide their children’s dental experiences in a positive and supportive way, aiming to help preventing early-onset dental fear or anxiety and promoting long-term positive attitudes toward dental visits and oral health. Targeted educational materials—such as booklets and videos offering psychological guidance for parents—are valuable tools that can be easily distributed online or through public and private dental sectors.

Longitudinal and controlled studies are needed in order to objectively evaluate the extent to which such feasible initiatives, subjectively perceived as useful by the targeted population, may influence children’s behavior and help avoid early onset of dental anxiety.

## Figures and Tables

**Table 1:** Demographic characteristics of the analyzed participants, childhood dental experiences, and attitudes toward children’s dental care.

Parameter	Value
**Section 1**	
**Parent—gender (female) (No., %)**	125 (88%)
**Parent—age (No., %)**	
18–25 years	3 (2.1%)
26–35 years	20 (14.1%)
36–45 years	74 (52.1%)
46–55 years	42 (29.6%)
>55 years	3 (2.1%)
**Number of children (No., %)**	
One	62 (43.7%)
Two	74 (52.1%)
Three	6 (4.2%)
**Child—age (mean** ± **SD, median (IQR))**	8.4 ± 5.17, 8 (5–11)
**Parent education (No., %)**	
Lower secondary (gymnasium)	2 (1.4%)
Secondary (high school)	12 (8.5%)
Post-secondary (college)	10 (7%)
Tertiary (university)	118 (83.1%)
**Section 2**	
**Negative childhood experiences (No., %)**	
Absent	68 (47.9%)
Present	63 (44.4%)
Don’t remember	11 (7.7%)
**Narration to children (No., %)**	
Absent	41 (65.1%)
Present	18 (28.6%)
Don’t remember	4 (6.3%)
**Dentist anxiety (No., %)**	
Not at all	55 (38.7%)
Low	55 (38.7%)
Neutral	8 (5.6%)
High	19 (13.4%)
Very high	5 (3.5%)
**Section 3**	
**Dental visit of children (No., %)**	134 (94.4%)
**Child—age—visit (mean** ± **SD, median (IQR))**	4.45 ± 2.3, 4 (3–6)
**Dental visit—reason (No., %)**	
Pain	8 (6.1%)
Check-up	72 (54.5%)
Cavity	32 (24.2%)
Other	20 (15.2%)
**Child—Negative experiences (No., %)**	12 (8.5%)
**Informing children before visit (No., %)**	121 (85.2%)
**Preferred dentist (No., %)**	
Pediatric dentist	104 (73.2%)
Same dentist as parents’	24 (16.9%)
Let children decide	8 (5.6%)
One closer to home	6 (4.2%)
**Child pain tolerance during treatment—normal (No., %)**	95 (66.9%)
**General anesthesia—ideal for pain control (No., %)**	
Highly disagree	56 (39.4%)
Disagree	29 (20.4%)
Neutral	34 (23.9%)
Agree	20 (14.1%)
Highly agree	3 (2.1%)

**Table 2:** Relationship between participants’ self-reported level of dental anxiety and: (a) their tendency to share negative childhood dental experiences with their children; (b) their tendency to prefer dental general anesthesia.

(a) Anxiety/Children narration (No., %) (N = 63)	Very low	Low	Neutral	High	Very high	*p* *
Absent	8 (88.9)	19 (63.3)	4 (100)	7 (46.7)	3 (60)	0.307 ϕc = 0.296
Present	1 (11.1)	10 (33.3)	0 (0)	5 (33.3)	2 (40)	
Don’t remember	0 (0)	1 (3.3)	0 (0)	3 (20)	0 (0)	
(b) Anxiety/General anesthesia as ideal (No., %) (N = 142)	Very low	Low	Neutral	High	Very high	*p* *
Highly disagree	23 (41.8)	25 (45.5)	4 (50)	4 (21.1)	0 (0)	0.124 ϕc = 0.209
Disagree	9 (16.4)	10 (18.2)	1 (12.5)	8 (42.1)	1 (20)	
Neutral	11 (20)	14 (25.5)	3 (37.5)	5 (26.3)	1 (20)	
Agree	9 (16.4)	6 (10.9)	0 (0)	2 (10.5)	3 (60)	
Highly agree	3 (5.5)	0 (0)	0 (0)	0 (0)	0 (0)	

* Fisher’s Exact Test, ϕc = Cramer’s V effect size.

**Table 3:** Perceived usefulness of information booklet.

		No.	%
Utility of information booklet	Not at all	3	2.1%
	Low	13	9.2%
	Neutral	23	16.2%
	High	63	44.4%
	Very high	40	28.2%
Recommendation to other parents	Highly disagree	0	0%
	Disagree	7	4.9%
	Neutral	5	3.5%
	Agree	53	37.3%
	Highly agree	77	54.2%

## Data Availability

The data presented in this study is available upon request from the corresponding authors.

## Abbreviations

The following abbreviations are used in this manuscript:

PD	Pediatric Dentistry
BG	Behavior Guidance

## References

[B1-behavsci-16-00620] Bagattoni S., Nascimben F., Biondi E., Fitzgibbon R., Lardani L., Gatto M. R., Piana G., Mattarozzi K. (2022). Preparing children for their first dental visit: A guide for parents. Healthcare.

[B2-behavsci-16-00620] Besiroglu-Turgut E., Kayaalti-Yuksek S., Bulut M. (2024). Evaluation of the relationship between dental anxiety and oral health status of mothers and their children. BMC Oral Health.

[B3-behavsci-16-00620] Dahlander A., Soares F., Grindefijord M., Dahllöf G. (2019). Factors associated with dental fear and anxiety in children aged 7 to 9 years. Dentistry Journal.

[B4-behavsci-16-00620] Dalsochio L., Montagner A. F., Tedesco T. K., Maske T. T., van de Sande F. H. (2025). Experiences and parents’ perceptions regarding dental interventions performed on their children: A qualitative systematic review. International Journal of Paediatric Dentistry.

[B5-behavsci-16-00620] Deshpande A., Jain A., Shah Y., Jaiswal V., Wadhwa M. (2002). Effectiveness of self-designed dental storybook as behaviour modification technique in 5–7 year-old children: A randomized controlled study. Journal of Indian Society of Pedodontics and Preventive Dentistry.

[B6-behavsci-16-00620] Doğan G. K., Polat Y., Özüdoğru S. (2025). Parental awareness and attitudes towards pediatric dentistry and children’s oral health. BMC Oral Health.

[B7-behavsci-16-00620] Emerson L. M., Ogielda C., Rowse G. (2019). A systematic review of the role of parents in the development of anxious cognitions in children. Journal of Anxiety Disorders.

[B8-behavsci-16-00620] Erdede O., Sari E., Uygur Külcü N., Sezer Yamanel R. (2022). Parents’ awareness of their children’s oral-dental health: Knowledge, attitudes and practices. Medicine Science|International Medical Journal.

[B9-behavsci-16-00620] Fox C., Newton J. T. (2006). A controlled trial of the impact of exposure to positive images of dentistry on anticipatory dental fear in children. Community Dentistry and Oral Epidemiology.

[B10-behavsci-16-00620] Freeman R. (2007). Viewing positive images of dentistry reduces anticipatory anxiety in children. Evidence-Based Dentistry.

[B11-behavsci-16-00620] Grisolia B. M., Dos Santos A. P. P., Dhyppolito I. M., Buchanan H., Hill K., Oliveira B. H. (2021). Prevalence of dental anxiety in children and adolescents globally: A systematic review with meta-analyses. International Journal of Paediatric Dentistry.

[B12-behavsci-16-00620] Kaushik M., Sood S. (2023). A systematic review of parents’ knowledge of children’s oral health. Cureus.

[B13-behavsci-16-00620] Marshman Z., Rodd H., Fairhurst C., Porritt J., Dawett B., Day P., Innes N., Vernazza C., Newton T., Ronaldson S., Cross L., Ross J., Baker S. R., Hewitt C., Torgerson D., Ainsworth H. (2023). The CALM trial protocol: A randomised controlled trial of a guided self-help cognitive behavioural therapy intervention to reduce dental anxiety in children. Trials.

[B14-behavsci-16-00620] Meshki R., Basir L., Alidadi F., Behbudi A., Rakhshan V. (2018). Effects of pretreatment exposure to dental practice using a smartphone dental simulation game on children’s dental anxiety: A preliminary double-blind randomized clinical trial. Journal of Dentistry.

[B15-behavsci-16-00620] Mittal M., Chopra R., Malhotra D., Dharmasya T., Gupta N., Lokade A., Atri M. (2025). Impact of dentistry modeling story on dental anxiety in 6–8-year-old children of industrial workers: A randomized controlled trial. Journal of Indian Society of Pedodontics and Preventive Dentistry.

[B16-behavsci-16-00620] Murshid E. Z. (2017). Effectiveness of a preparatory aid in facilitating oral assessment in a group of Saudi children with autism spectrum disorders in Central Saudi Arabia. Saudi Medical Journal.

[B17-behavsci-16-00620] Nowak A. J., Casamassimo P. S. (2002). The dental home: A primary care oral health concept. The Journal of the American Dental Association.

[B18-behavsci-16-00620] Oliveira M. A., Vale M. P., Bendo C. B., Paiva S. M., Serra-Negra J. M. (2017). Influence of negative dental experiences in childhood on the development of dental fear in adulthood: A case-control study. Journal of Oral Rehabilitation.

[B19-behavsci-16-00620] Ollendick T. H., King N. J. (1991). Origins of childhood fears: An evaluation of Rachman’s theory of fear acquisition. Behaviour Research and Therapy.

[B20-behavsci-16-00620] Olsson A., Nearing K. I., Phelps E. A. (2007). Learning fears by observing others: The neural systems of social fear transmission. Social Cognitive and Affective Neuroscience.

[B21-behavsci-16-00620] Padung N., Singh S., Awasthi N. (2022). First dental visit: Age, reasons, oral health status, and dental treatment needs among children aged 1 month to 14 years. International Journal of Clinical Pediatric Dentistry.

[B22-behavsci-16-00620] Porritt J., Buchanan H., Hall M., Gilchrist F., Marshman Z. (2013). Assessing children’s dental anxiety: A systematic review of current measures. Community Dentistry and Oral Epidemiology.

[B23-behavsci-16-00620] Porritt J., Rodd H., Morgan A., Williams C., Gupta E., Kirby J., Creswell C., Newton T., Stevens K., Baker S. (2016). Development and testing of a cognitive behavioral therapy resource for children’s dental anxiety. JDR Clinical & Translational Research.

[B24-behavsci-16-00620] Raadal M., Strand G. V., Amarante E. C., Kvale G. (2002). Relationship between caries prevalence at 5 years of age and dental anxiety at 10. European Journal of Paediatric Dentistry.

[B25-behavsci-16-00620] Rachman S. (1977). The conditioning theory of fear-acquisition: A critical examination. Behaviour Research and Therapy.

[B26-behavsci-16-00620] Ramesh R., Sathyaprasad S., Nandan S., Havaldar K. S., Antony A. (2024). Assessment of preappointment parental counseling on dental fear and anxiety in children in pedodontic dental operatory: A randomized controlled trial. International Journal of Clinical Pediatric Dentistry.

